# A comparison of burn related injuries following the natural disaster Super Storm Hurricane Sandy to the National Burn Repository of the American Burn Association

**DOI:** 10.1186/s41038-015-0025-5

**Published:** 2016-01-13

**Authors:** Michael Kalina, Grigoriy Malyutin, Michael L. Cooper

**Affiliations:** Emergency Surgery, Staten Island University Hospital, 256 Mason Avenue, Suite C, Staten Island, NY 10305 USA

**Keywords:** Natural disaster, Super Storm Hurricane Sandy, Burn

## Abstract

**Background:**

Burn related injuries from natural disasters are not well described and natural disasters are not identified as an etiology of burn injury in the National Burn Repository (NBR) of the American Burn Association. The natural disaster Super Storm Hurricane Sandy had devastating effects. Our goal was to detail the burn related injuries following this natural disaster and to compare the data to the NBR.

**Methods:**

This was a retrospective chart review of thirty four patients who sustained burn related injuries following Super Storm Hurricane Sandy (SSHS) and were managed at Staten Island University Hospital Burn Center. Institutional Review Board approval was obtained. Data variables included age, gender, race, past medical history (PMHx), burn type, percentage total body surface area (%TBSA), hospital length of stay (HLOS), and mortality. We compared data from SSHS to the 2003-2013 NBR. Categorical data were summarized using frequency counts, percentages and Clopper-Pearson 95 % confidence interval for proportion. Continuous outcome data were summarized by descriptive statistics. Data analyses performed with SAS® System Version 9.3 (SAS Institute Inc., Cary, NC) and *p* < 0.05 was significant.

**Results:**

In the SSHS group, average age was 36 + 24 years, range 1-80 years, and 44.1 % were males (15/34, 95 % CI: 27.2 - 62.1). Caucasians comprised 58.8 %, (20/34, 95 % CI: 40.7, 75.4) and 73.5 % had no PMHx (25/34, 95 % CI: 55.6, 87.1). The most common burn type was scald, 55.9 %, (19/34, 95 % CI: 37.9, 72.8) and %TBSA ranged 1 %–47 %, average of 7 % + 12 %. The average HLOS was 13 + 26 days, range of 1–113 days. Mortality was 2.9 % (1/34, 95 % CI: 0.07–15). In comparison, the NBR reported an average age of 32 years and 69 % were males. Caucasians comprised 59.1 %. The most common burn type was flame, 43.2 % and the %TBSA ranged 1 %–9.9 %. HLOS ranged 8.4–10.2 days and mortality was 3.4 %.

**Conclusion:**

We conclude that burn related injuries following a natural disaster differ as compared to those most commonly reported in the NBR.

## Background

According to the Glossary of Humanitarian Terms from the World Health Organization, the definition of a natural disaster is an event brought about by natural hazards that can seriously affect the society, economy and/or infrastructure of a region. A natural hazard is a natural process or phenomenon that may cause the loss of life or injury, property damage, social and economic disruption or environmental degradation. Natural hazards can include earthquakes, volcanic activity, landslides, tropical cyclones, other severe storms, tornadoes, flooding, wildfires, drought, sand/dust storms, and infestations [1]. The natural disaster Super Storm Hurricane Sandy (SSHS) began as a cyclone, intensified into a category 3 hurricane during its course, and made landfall in New Jersey as a tropical storm. The impact and storm surge of SSHS had catastrophic effects along the New Jersey and New York coastlines with U.S. damage estimates near 50 billion dollars. Direct deaths, those persons who drowned in storm surge, were estimated at 147 fatalities. The devastation was widespread in Staten Island, New York with 21 fatalities or indirect deaths, those occurring from secondary factors such as heart attacks, motor vehicle accidents, and burn related injuries. The damage was so severe in Staten Island that according to the Tropical Cyclone Report from the National Hurricane Center, Staten Island was referred to as “Ground Zero” for damage in New York City [2, 3]. Staten Island University Hospital (SIUH) is a New York State Verified Level 1 Trauma Center and the only New York State accredited Burn Center on Staten Island. Although the hospital incurred significant structural damage in some areas from the storm, it remained open, functional, and a number of victims from SSHS, many of whom had burn related injuries, were cared for. This study is our review of the burn related injuries seen at Ground Zero following the natural disaster, SSHS. As no subgroup analysis of burn victims from a natural disaster exists within the National Burn Repository, our goal was to compare our findings to those most commonly reported within the repository.

## Methods

This was a retrospective chart review of patients who sustained burn related injuries from SSHS. All patients were managed at Staten Island University Hospital Burn Center, between October 29, 2012 and November 29, 2012. Institutional Review Board approval was obtained. Data variables acquired included age, gender, race, past medical history, burn type, total body surface area of burn, hospital length of stay, need for debridement, and mortality. We compared the burn related injuries from SSHS to those reported in the 2003-2013 National Burn Repository (NBR) of the American Burn Association. Frequency distribution or descriptive statistics for demographic and baseline disease characteristics were presented for all patients. Categorical data were summarized using frequency counts, percentages and Clopper-Pearson 95 % confidence interval for proportion. Continuous outcome data were summarized by descriptive statistics such as mean, standard deviation and 95 % confidence interval. We compared the burn related injuries from SSHS to the NBR using two-sided 95 % confidence intervals for a single proportion or for a single mean. All statistical tests were 2-sided and *p* ≤ 0.05 was statistically significant. Data analyses were performed using the SAS® System Version 9.3 (SAS Institute Inc., Cary, NC).

## Results

Thirty four patients were included in the study. The average age was 36 ± 24 years, range 1-80 years, and there were 44.1 % males (15/34, 95 % CI: 27.2 – 62.1). Caucasians were the most common race seen in the SSHS group, 58.8 %, (20/34, 95 % CI: 40.7, 75.4) and most SSHS patients had no significant past medical history 73.5 % (25/34, 95 % CI: 55.6, 87.1). In comparison, the 2003-2013 NBR summary report revealed that the average age was 32 years old. There was no difference in age between groups. There were 69 % males in the NBR, which was higher than the percentage of males in the SSHS group. The most common race in the NBR was Caucasian, 59.1 %, which was similar to the SSHS group (Table 1). In the SSHS group, the most common type of burn related injury was scald burns, 55.9 %, (19/34, 95 % CI: 37.9, 72.8), as compared to the NBR where the most common burn type was flame, 43.2 %. The range of percentage total body surface area (%TBSA) of burn was 1 %-47 % with an average %TBSA of 7 % ± 12 % in the SSHS group. The NBR, however, reported that the most common range of %TBSA was between 1-9.9 %, accounting for 74.8 % of all burns reported from 2003-2013. Most SSHS patients did not require debridement, 91.2 % were managed with topical therapy only (Table 2). The average hospital length of stay for the SSHS group was 13 ± 26 days, with a range of 1-113 days; whereas, the NBR reported a range of hospital length of stay from 8.4-10.2 days. Mortality was 2.9 % (1/34, 95 % CI: 0.07 – 15) in the SSHS group and this was not different that the reported mortality of 3.4 % in NBR patients (Table 3).

**Table 1:** Demographics

	SSHS	NBR	CLOPPER-PEARSON
AGE (YEARS)	36 + 24 (RANGE 1-80)	32	N/A
GENDER (MALES)	44.1 %	69 %	0.38-0.73
RACE (CAUCASIAN)	58.8 %	59.1 %	0.41-0.75
RACE (BLACK)	11.8 %	19.5 %	0.03-0.27
RACE (OTHER)	29.4 %	21.4 %	0.15-0.47
NO PMHx	73.5 %	N/A	0.55-0.87
DM	5.9 %	N/A	0.01-0.19
HTN	23.5 %	N/A	0.11-0.41
CAD	5.9 %	N/A	0.01-0.19
CKD	2.9 %	N/A	0.00-0.15

*SSHS = Super Storm Hurricane Sandy; NBR = National Burn Repository; CLOPPER PEARSON = 95 % confidence interval; H-LOS = Hospital Length of Stay* ; *PMHx = Past Medical History; DM = Diabetes; HTN = Hypertension; CAD = Coronary Artery Disease; CKD = Chronic Kidney Disease; N/A= not applicable or unable to obtain*

**Table 2:** Burn total body surface area, type, and treatment

	SSHS	NBR	CLOPPER-PEARSON
% TBSA	7 % + 12 % (RANGE 1–47 %)	1–9.9 % (RANGE)	N/A
SCALD	55.9 %	34 %	0.38–0.73
FLAME	14.7 %	43.2 %	0.05–0.31
CONTACT	17.6 %	9 %	0.07–0.34
CHEMICAL	5.9 %	3 %	0.01–0.19
ELECTRICAL	5.9 %	4 %	0.01–0.19
TOPICAL	91.2 %	N/A	0.76–0.98
DEBRIDMENT	8.8 %	N/A	0.02–0.24

*SSHS = Super Storm Hurricane Sandy; NBR = National Burn Repository; CLOPPER PEARSON = 95 % confidence interval; % TBSA = Percentage of Total body Surface Area burned; TOPICAL = local wound dressings with the use of topical ointments including bacitracin and silvadene ; N/A = not applicable or unable to obtain*

**Table 3:** Outcomes

	SSHS	NBR	CLOPPER-PEARSON
ADMITTED	58.8 %	N/A	0.41–0.75
DISCHARGED	41.2 %	86 %	0.25–0.59
H-LOS (DAYS)	13 + 26 (RANGE 1–113)	8.4–10.2 (RANGE)	N/A
MORTALITY	2.9 %	3.4 %	0.00–0.15

*SSHS = Super Storm Hurricane Sandy; NBR = National Burn Repository; CLOPPER PEARSON = 95 % confidence interval; ADMITTED = Admitted to the hospital for in-patient care; DISCHARGED = Discharged from the Emergency Department; H_LOS = Hospital Length of Stay; N/A = not applicable or unable to obtain*

## Discussion

There are few reports of burn related injuries derived from natural disasters in the literature. Much of the existing literature includes burn injuries derived from crime, terrorism, warfare, and accidents. Mahoney and colleagues in 2005 detailed the burn related injuries from a nightclub fire in Rhode Island. The study illustrated the characteristics of 439 burn victims, of which 47 patients were admitted to Rhode Island Hospital. They found an average age of 31.9 ± 6.4 years, an average % TBSA of 18.8 % ± 13.8 %, and an average hospital length of stay of 21.5 ± 18.6 days. The study did not differentiate the types of burns encountered [4]. In 2011, Kooij et al chronicled the care of 89 burn victims at a rural hospital in Kenya, after a fuel tanker explosion. Eighty-six (97 %) were men with a median age of 25 years. 45 patients died within 1 week and had a total body surface area burned of 80 % as compared with 28 % for survivors. They concluded that in areas where referral to tertiary centers is not possible, district hospitals should have mass disaster plans that involve burn related victims [5]. In this study, we were able to detail the burn related injuries following the natural disaster SSHS.

NBR of the American Burn Association (ABA) began over 50 years ago and is a database that encompasses the preceding ten-years of cumulative experience from the contributing burn centers. The NBR data set currently has over 175,000 entries from 91 burn centers in the United States, 4 burn centers from Canada and 2 burn centers from Sweden. The database stratifies burn related injuries by etiology, age, gender, and survival and includes hospital length of stay and disposition of survivors. The burn center at Staten Island University Hospital, Ground Zero for SSHS, is a New York State verified Burn Center but does not hold American Burn Association certification and does not contribute to the NBR. As of 2012 there were 123 self-designated burn care facilities in the United States, 12 of who are in New York State. As of 2015, 62 burn centers are designated by the American Burn Association, of which only 1 center is ABA certified in New York State. Although the NBR catalogues a wealth of burn injury data, not all centers contribute. Also, the NBR does not specifically delineate natural disaster as an etiology of burn related injuries and it is lacking in risk adjustment, standardized definitions of burn etiology, and in its ability to assist in benchmarking the performance of individual centers [6]. Our data suggest that there is a difference in the types of burns seen in victims following a natural disaster as compared to the existing data within the NBR; however the reason for these differences is unclear. The most common type of burn in SSHS was scald in comparison to flame in the NBR. The World Health Organization definition of scald burn is when the skin or other tissue is being destroyed by a hot liquid; whereas, the definition of a flame burn is when the skin or other tissue is destroyed by flame [7]. Most scald burn injuries happen in the home, in connection with the preparation or serving of hot food or beverages, or from exposure to hot tap water in bathtubs or showers. Severe scalds also occur in the workplace, typically when pipes or valves fail while carrying or regulating the flow of steam [8]. There were widespread power outages and losses of hot water and heat after SSHS. Scald injuries may have been more prevalent after this natural disaster because of the damage to hot water pipes and hot water heaters and the use of portable heating sources to cook food and boil water. Without benchmarking and without the ability to reliably compare burn etiologies or burn types seen in a natural disaster and the NBR, the true impact of these differences remains to be realized. Future investigations into these discrepancies as well as a better delineation of burn etiology to include natural disasters in the NBR may lead to improved data interpretation and burn disaster management protocols ultimately leading to improved patient care.

## Conclusion

We conclude that the burn related injuries following the natural disaster Super Storm Hurricane Sandy differ with respect to burn type as compared to those most commonly reported in the National Burn Repository of the American Burn Association.
